# The Effect of Codon Mismatch on the Protein Translation System

**DOI:** 10.1371/journal.pone.0148302

**Published:** 2016-02-03

**Authors:** Dinglin Zhang, Danfeng Chen, Liaoran Cao, Guohui Li, Hong Cheng

**Affiliations:** 1 Laboratory of Molecular Modeling and Design, State Key Laboratory of Molecular Reaction Dynamics, Dalian Institute of Chemical Physics, Chinese Academy of Sciences, Dalian, Liaoning, 116023, China; 2 Dalian City Fisherles Technical Extension Station, Dalian, Liaoning, 116025, China; 3 Shanghai Key Laboratory of Molecular Andrology, State Key Laboratory of Molecular Biology, Institute of Biochemistry and Cell Biology, Shanghai Institutes for Biological Sciences, Chinese Academy of Sciences, Shanghai, 200031, China; Pohang University of Science and Technology, REPUBLIC OF KOREA

## Abstract

Incorrect protein translation, caused by codon mismatch, is an important problem of living cells. In this work, a computational model was introduced to quantify the effects of codon mismatch and the model was used to study the protein translation of Saccharomyces cerevisiae. According to simulation results, the probability of codon mismatch will increase when the supply of amino acids is unbalanced, and the longer is the codon sequence, the larger is the probability for incorrect translation to occur, making the synthesis of long peptide chain difficult. By comparing to simulation results without codon mismatch effects taken into account, the fraction of mRNAs with bound ribosome decrease faster along the mRNAs, making the 5’ ramp phenomenon more obvious. It was also found in our work that the premature mechanism resulted from codon mismatch can reduce the proportion of incorrect translation when the amino acid supply is extremely unbalanced, which is one possible source of high fidelity protein synthesis after peptidyl transfer.

## Introduction

Understanding of the gene translation process is important for human health[1–3], biotechnology [4–6] and evolution[3,4,7,8]. In recent years a number of technologies have been developed to characterize different features related to the gene translation and multiple roles of the coding sequence have been proposed. Recent studies suggested that the order of codons along the mRNA plays an important role in determining translation efficiency[4,9–11]. It was suggested that there is weak folding of mRNA molecule in the region surrounding the start codons[9–16], and endogenous genes tend to perform strong mRNA folding in the region after the start codon[10,17,18], which can improve the fidelity of translation initiation[10,17,19,20]. It was also suggested that the first 30–50 codons at the beginning of the open reading frame (ORF) tended to be recognized by tRNA species with lower intracellular abundance[6,21,22], resulting in slower ribosomal elongation speed in this region[6,23,24]. Fast initiation of short genes also causes a 5’ ribosomal ramp[14]. However, this process is still enigmatic with contradicting conclusions in different studies. Although there are many publications show that the codon sequence is one reason of the 5’ end ramp, other research suggested that the ramp of 5’ end is caused primarily by faster initiation in short genes, rather than by the ordering of codons within each gene[14]. In this paper, we proposed that the ribosome premature is another causeof5’ ramp.

Translation of mRNA has been studied by a variety of computational models based on the totally asymmetric simple exclusion process, justifying the role of codon ordering in determining spatial patterns of ribosomes along mRNAs[18,25]. Such models were built based on constant, inexhaustible supplies of amino acids, free ribosomes and free tRNAs in the cell. A more realistic alternation, the “Whole cell”[14] was developed to investigate the gnome scale gene translation properties. This model tracks all ribosomes and tRNAs in a cell—each of which is either freely diffusing or bound to a specific mRNA molecule at a specific codon position at any time point. Transition rates among states are parameterized in seconds so that the model describes the dynamics of translation in real time. Unlike many other models of translation, which treat each mRNA molecule in isolation and assume an inexhaustible supply of free ribosomes that initiate the message at a constant rate, “Whole cell” model keeps track of every tRNA, mRNA, and ribosome molecule in the cell simultaneously. But codon mismatch effects, leading to premature, especially under unbalanced starvation conditions[26,27], was still ignored in the “Whole cell” model. Our model are based on the “Whole cell” model, and focus on a previously ignored problem: the effect of codon mismatch and translation premature. From the simulation result of our model, the premature is one reason for the 5’ ramp and cannot be ignored. Although the chance of codon mismatch at each codon is low[28], it can be accumulated along the long mRNA sequence. In order to investigate the codon mismatch effects in translation processes[29–32], we built a model to study the translation performance under two different conditions: balanced and unbalanced amino-acid supplies.

## Methods

### Model description

**Fig 1:**
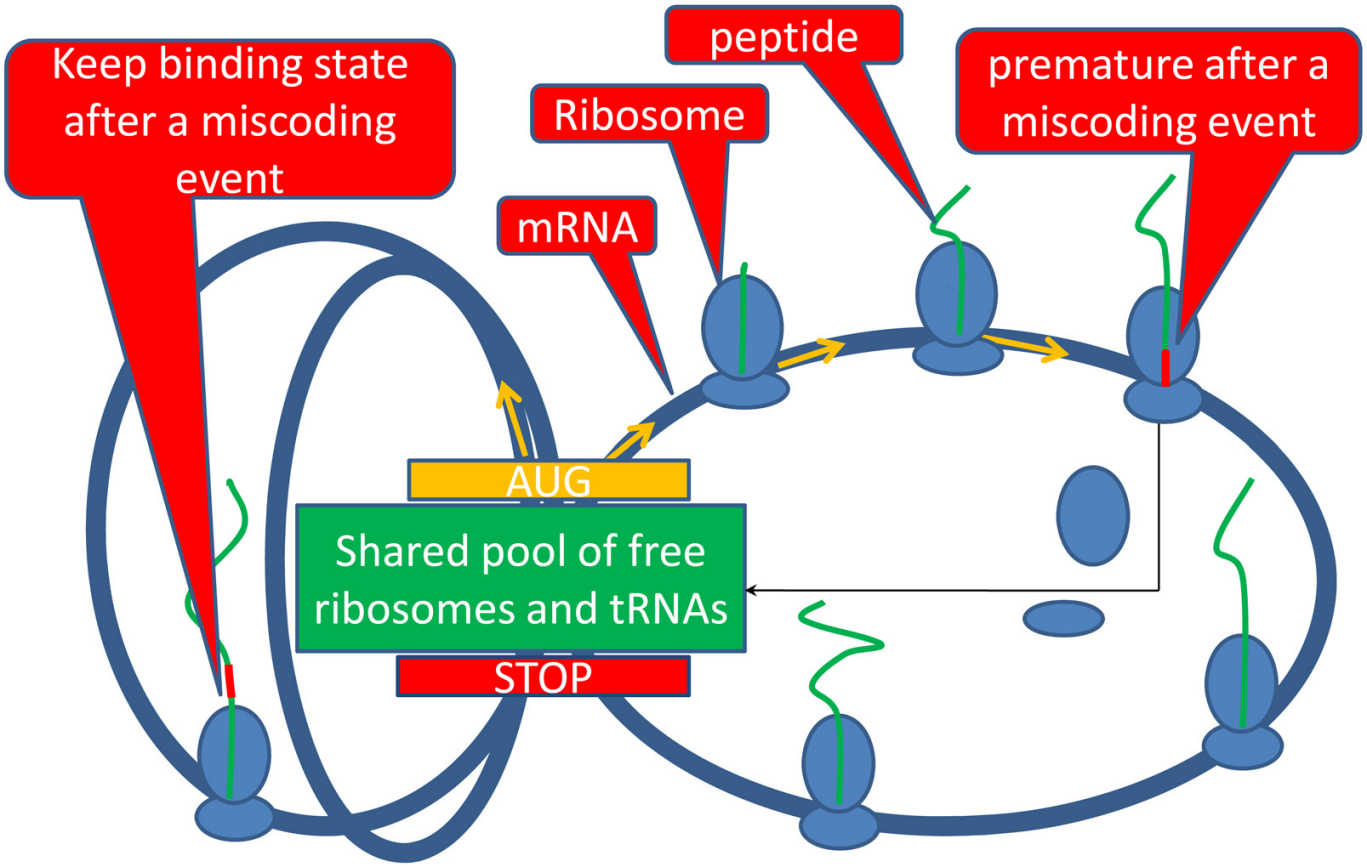
Translation schematic diagram. When a codon mismatch occurs, the ribosome will either abort or continue to translate the left codons. When the ribosome aborts, all the work has been done will become invalid and the ribosome will return to the free pool, while some ribosomes can still reach the ending codons with incorrect peptides, which will decrease translation efficiency.

Our model takes the premature event into account (Fig 1). Consider a set of mRNA sequences and each has *N*_*i*_ sites (*i* is the index of sequence) which can either be occupied by a ribosome or be empty, ribosomes can transfer between different sites according to the following rules: given a movable ribosome randomly, if its current position *j* (*j*, 1 ≤ *j* ≤ *N*_*i*_) is between 1 and *N*_*i*_− 1, then the ribosome on site *j* will move to site *j* + 1.

**Fig 2:**
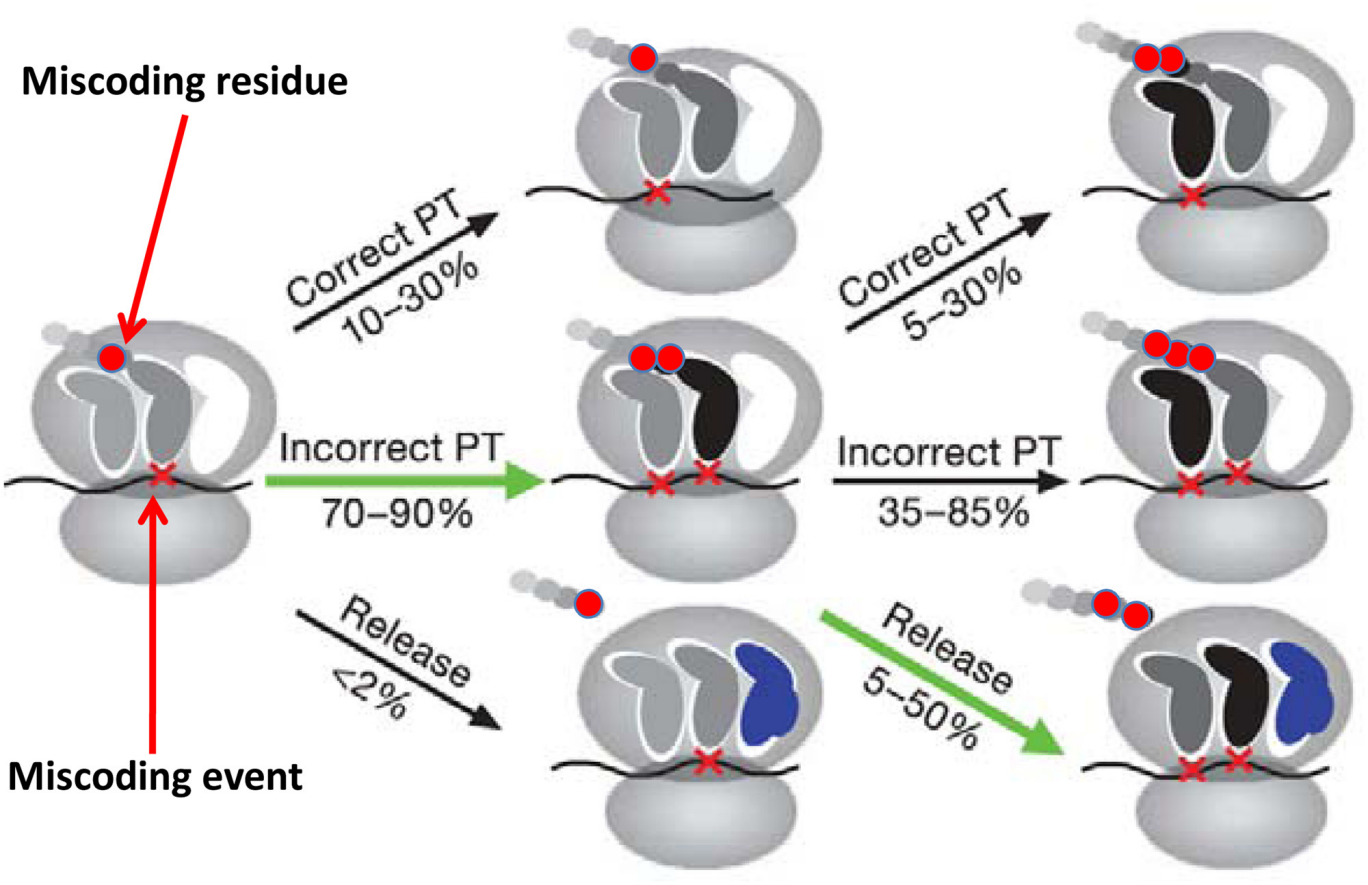
An initial miscoding event results in an overall drop in yield of full-length peptides. (adapted from nature. 2009, 457(8):161, with permission from NATURE) Proposed model for the events after a miscoding event with the steps contributing to the quality control described here highlighted by green arrows. PT, peptidyl transfer.

In this process, codon mismatch can occur based on a predefined probability. For mRNA *i*, codon *j*, the codon mismatch probability *p*_*j_mis*_ can be calculated based on the number of available aminoacyl tRNAs (Eqs 1–5). Here *p*_*mis_based*_ is the average mismatch probability under normal conditions, which is available from experimental results[28]; *p*_*relative_mis_aa*_ is the average probability of mismatch for the current codon under normal amino acid supply conditions; *p*_*relative_match_aa*_ is the average match probability for the current codon under normal amino acid supply conditions; *p*_*mis_abort*_ is the total premature probability for a ribosome when a codon mismatch occurs. If a non-cognate amino acyl tRNA is incorporated, the peptide chain will lose specificity in the A site of the ribosome and the propagation of this error may result in premature of the peptide[28,33]. From Fig 2 [28], we can see that when a codon mismatch occurs, the premature probability for a ribosome is rather high. Because the probability of continuing an incorrect translation will be significantly reduced, the premature termination is assumed to occur immediately after the first mismatch with the defined probability in this model.

$$p_{relative\_mis\_aa}=\frac{p_{mis\_based}}{61}$$ (1)

$$p_{relative\_match\_aa}=1-p_{mis\_based}$$ (2)

**For the current codon *j*, the index of matched aminoacyl tRNA type is *t*; *x* is the set of matched aminoacyl tRNA;** *p*_*j_total_relative_mis*_ **is the probability of mismatch and** *p*_*j_total_relative_match*_ is the probability of match.

$$p_{j\_total\_relative\_mis}=\overset{}{\underset{t\notin x_{}}{\sum }}T_{t}^{f}p_{relative\_mis\_aa}$$ (3)

$$p_{j\_total\_relative\_match}=\overset{}{\underset{t\in x_{}}{\sum }}T_{t}^{f}p_{relative\_match\_aa}$$ (4)

$$p_{j\_mis}=\frac{p_{j\_total\_relative\_mis}}{p_{j\_total\_relative\_match}+p_{j\_total\_relative\_mis}}$$ (5)

$$p_{j\_match}=\frac{p_{j\_total\_relative\_match}}{p_{j\_total\_relative\_match}+p_{j\_total\_relative\_mis}}$$ (6)

Here $T_{t}^{f}$ is the number of free aminoacyl tRNAs of type *t*. For a movable ribosome on site *j*, it has three possible moves. The probability of the ribosome to move to the next site correctly is *p*_*j_match*_; the probability for it to move to the next position, but performing incorrect translation is *p*_*j_mis*_ × (1 –*p*_*mis_abort*_); and the probability of a premature termination is *p*_*j_mis*_ × *p*_*mis_abort*_.

A reasonable estimation of the initiation rate under various conditions is a key problem. Here we assume that the initiation rate is the bottleneck of translation rate because the average number of ribosomes on mRNA sequences is small, indicating that the transfer between different sites should be much faster than the initiation[18,24,34]. Here we infer that the initiation rate is determined by the supply of free Methionyl-tRNAs and ribosomes (Eqs 9 and 10).

Initiation rate for one mRNA of gene *i*:

$$if(M>R^{f}):\rho_{i}=\frac{R^{f}p_{i}}{\tau_{r}N_{r}}$$ (7)

$$if(M<=R^{f}):\rho_{i}=\frac{Mp_{i}}{\tau_{r}N_{r}}$$ (8)

Total initiation rate:

$$if(M>R^{f}):\rho^{t}=\overset{n}{\underset{i=1}{\sum }}\frac{R^{f}f_{i}A_{i}p_{i}}{\tau_{r}N_{r}}$$ (9)

$$if(M<=R^{f}):\rho^{t}=\overset{n}{\underset{i=1}{\sum }}\frac{Mf_{i}A_{i}p_{i}}{\tau_{r}N_{r}}$$ (10)

*f*_*i*_ is the fraction of mRNAs of gene *i* that can be initialized. *A*_*i*_ is the number of mRNAs of type *i*. *p*_*i*_ is the gene-specific initiation probability[14], *D*_*r*_ is the diffusion coefficient of ribosomes. *D*_*t*_ is the diffusion coefficient of tRNAs, *τ*_*r*_ and *τ*_*t*_ are the characteristic times of ribosomes and tRNAs[14], *R*^*f*^ is the number of free ribosomes, *M* is the number of free Methionyl-tRNAs. Because the mismatched aminoacyl tRNAs also have a certain chance to be accepted in the translation especially with an unbalanced amino acid supply, a transfer rate is introduced.

Transfer rate for one ribosome on codon:

$$\varepsilon_{j}=\frac{C_{j}^{f}\omega_{j}s}{\tau_{t}N_{t}}$$ (11)

Total transfer rate:

$$\varepsilon^{t}=\overset{61}{\underset{j=1}{\sum }}\frac{R_{j}^{b}C_{j}^{f}\omega_{j}s}{\tau_{t}N_{t}}$$ (12)

$$C_{j}^{f}=\overset{61}{\underset{k=1}{\sum }}p_{k\_j}\times T_{k}^{f}$$ (13)

If the anticodon *k* does not match codon *j*:

$$p_{k\_j}=\frac{p_{mis\_based}}{61}$$ (14)

If the anticodon *k* matches codon *j*:

$$p_{k\_j}=1-p_{mis\_based}$$ (15)

*ω*_*j*_ is the wobble parameter, *s* is tRNA competition coefficient, $T_{k}^{f}$ is the number of free tRNAs of type *k*. This model can also simulate the process that does not count codon mismatchby setting *p*_*mis_based*_ = 0.

### Simulation Setup Details

To investigate how protein production is affected by stress, we simulated translation under conditions of balanced and unbalanced amino-acid supply conditions. We modeled the stress of a particular amino acid by changing the abundance of its (charged) cognate tRNAs by 2^x^ folds[14]. Here 2^x^ is the supply coefficient. The package used for the simulations can be download from ftp://159.226.238.166/pub/. It was written in C++ and can be compiled under centos. Based on the package of “Whole cell”[14], the codon mismatch and premature features were added.

**Condition 1**: a balanced amino acid supply condition in which the amount of each aminoacyl tRNAs is multiplied by the same supply coefficient 2^x^. When *x* = 0, the system is in normal amino acid supplycondition[14].

**Condition 2**: an unbalanced amino acid supply condition. Here we take Arg imbalance as an example to investigate the performance of the translation system. So only the amount of Arginyl-tRNA is multiplied by a supply coefficient 2^x^.

**Condition 3**: random starvation condition that all amino-acid supplies are randomly modified, different amino acids have different coefficients less than one. In fact this condition is also an unbalanced condition.

**Normal amino acid supply condition**: the ratio of amino acid supply is defined with the relative copy numbers of charged aminoacyl tRNAs (Table 1) [14].

**Table 1:** The relative copy numbers of aminoacyl tRNAs.

aa	code	anti code	trna copy number	aa	code	anti code	trna copy number	aa	code	anti code	trna copy number
A	GCA	TGC	5	K	AAA	TTT	7	R	CGG	CCG	1
A	GCC	AGC	11	K	AAG	CTT	14	R	CGT	ACG	6
A	GCG	TGC	5	L	CTA	TAG	3	S	TCA	TGA	3
A	GCT	AGC	11	L	CTC	GAG	1	S	TCC	AGA	11
C	TGC	GCA	4	L	CTG	TAG	3	S	TCG	CGA	1
C	TGT	GCA	4	L	CTT	GAG	1	S	TCT	AGA	11
D	GAC	GTC	16	L	TTA	TAA	7	S	AGC	GCT	2
D	GAT	GTC	16	L	TTG	CAA	10	S	AGT	GCT	2
E	GAA	TTC	14	M	ATG	CAT	10	T	ACA	TGT	4
E	GAG	CTC	2	N	AAC	GTT	10	T	ACC	AGT	11
F	TTC	GAA	10	N	AAT	GTT	10	T	ACG	CGT	1
F	TTT	GAA	10	P	CCA	TGG	10	T	ACT	AGT	11
G	GGA	TCC	3	P	CCC	AGG	2	V	GTA	TAC	2
G	GGC	GCC	16	P	CCG	TGG	10	V	GTC	AAC	14
G	GGG	CCC	2	P	CCT	AGG	2	V	GTG	CAC	2
G	GGT	GCC	16	Q	CAA	TTG	9	V	GTT	AAC	14
H	CAC	GTG	7	Q	CAG	CTG	1	W	TGG	CCA	6
H	CAT	GTG	7	R	AGA	TCT	11	Y	TAC	GTA	8
I	ATA	TAT	2	R	AGG	CCT	1	Y	TAT	GTA	8
I	ATC	AAT	13	R	CGA	ACG	6				
I	ATT	AAT	13	R	CGC	ACG	6				

In our simulation, *p*_*mis_abort*_ was set to be 0.5, *p*_*mis_based*_ was set to be 0.001[28]. 3,795 genes and 60,000 mRNAs were adopted from S. cerevisiae[14]. To investigate the effects of mistranslation, we studied the translation under three conditions: Condition 1, Condition 2and Condition 3 as previously mentioned. Equilibrations in all simulation systems were achieved in the first 2x10^9^ steps, followed by another 100 seconds of simulation for data collection.

## Results and Discussions

### Incorrect translation proportion is high under unbalanced conditions

There will be 2 types of productions: one is the **correct translation production** which is the correct translation of the whole codon sequence. The second is **incorrect translation production** in which the peptide is synthesized with codon mismatch or is shorter than the correct peptide. The fraction of the three fractions were got from the simulation result and are defined as the following:

$$fraction\_premature\_len=\frac{total\_length\_of\_released\_premature\_chains}{total\_length\_of\_released\_chains}$$ (16)

$$fraction\_unabort\_len=\frac{total\_length\_of\_released\_unabort\_incorrect\_chains}{total\_length\_of\_released\_chains}$$ (17)

$$fraction\_incorrect\_len=\frac{total\_length\_of\_released\_incorrect\_chains}{total\_length\_of\_released\_chains}$$ (18)

**Fig 3:**
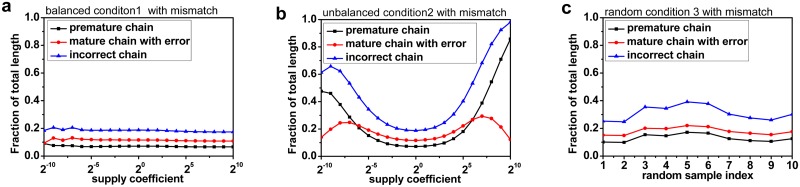
The relationship between incorrect translation proportion and amino acid supply. In Fig 3a, 3b and 3c, the vertical axis shows the total length fraction of the three kinds of released peptide chains which are the premature chains, mature chains with error, and the incorrect chains. In Fig 3a and 3b, the horizontal axis shows the amino acids supply coefficient. So in Fig 3a, all amino acid numbers were multiplied by the same coefficient, and in Fig 3b, only the number of Arg was multiplied by the coefficient while the number of other amino acids were kept unchanged. In Fig 3c, 10 simulations were run under Condition 3 independently and the horizontal axis shows the simulation index. The result shows that stress of unbalanced amino acid supply can lead to incorrect translation.

Compared to the simulation under Condition 1, the fraction of incorrect translation under unbalanced conditions is relatively high (Fig 3). The fraction of incorrect translation is minor and does not perform large fluctuations under Condition 1, while under Condition 2, the fraction of incorrect translation increases with the stress of unbalanced amino acid supply. Under Condition 3, all the 10 fractions of incorrect translation are higher than the normal conditions. This is an evidence of the premature termination mechanism, which can contribute to the reduction of incorrect production when the supply of amino acid is extremely unbalanced.

**Fig 4:**
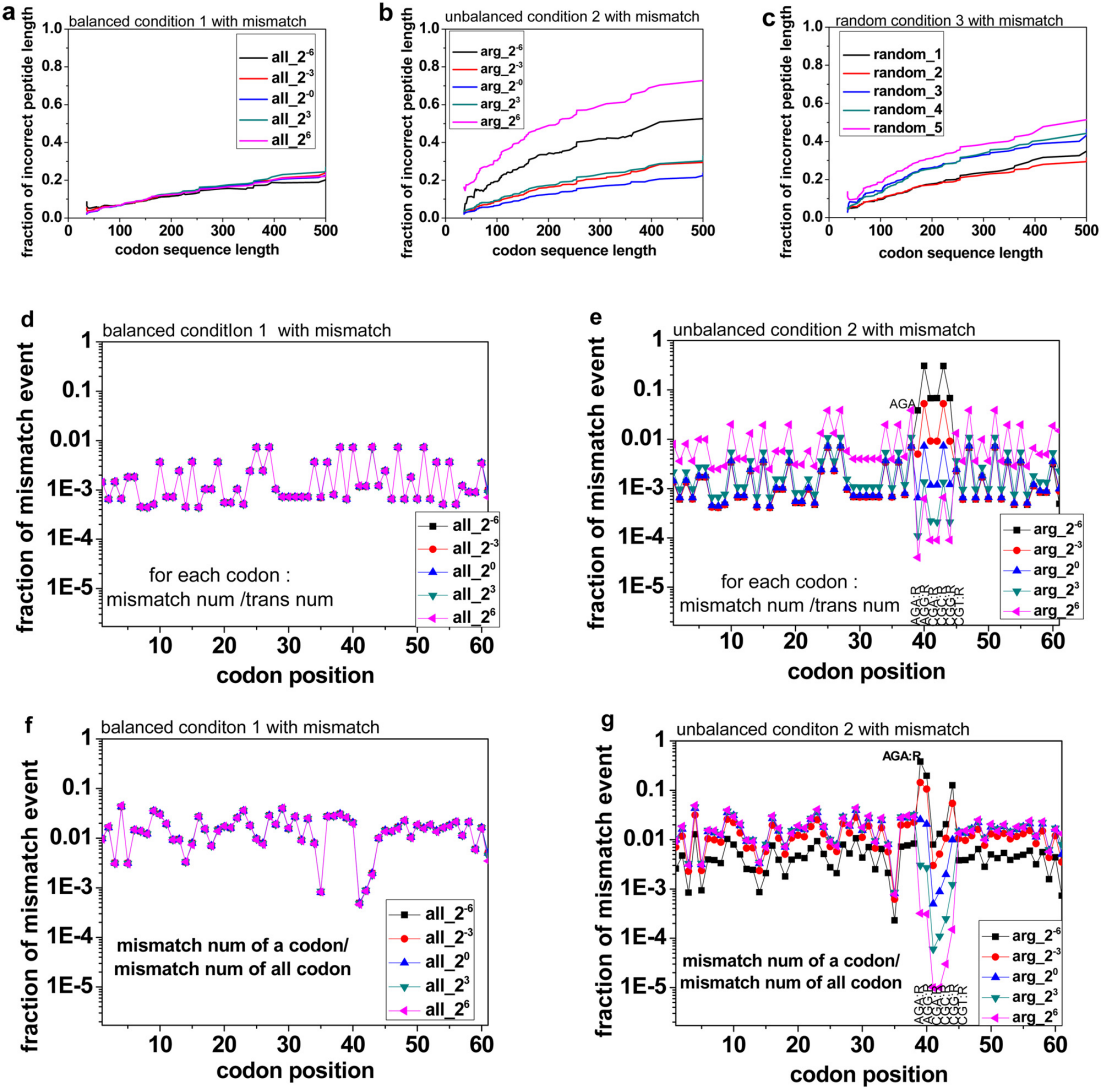
Incorrect translation proportion increases with codon sequence length. In Fig 4a, 4b and 4c, some of mRNAs were not translated for enough times, which could have a significant effect on the final result. So for each simulation result, the mRNAs were sorted with the correctly translated times, and the top 100 were selected. The vertical axis shows the total length fraction of the incorrect chains. The horizontal axis shows the codon sequence length. In Fig 4a and 4b, the incorrect translation fraction is plotted against the codon sequence length with 5 different supply coefficients; In Fig 4c, there are 5 curves for each simulation, different charged aminoacyl tRNA coefficient was randomly set less than 1; Fig 4d and 4e show the fraction of mismatch events on a codon, which is equal to the number of mismatch events on the codon divided by the number of all events on the same codon. Fig 4f and 4g show the fraction of mismatch events of a codon, which is equal to the number of mismatch events on the codon divided by the number of mismatch events on all codons.

Although the chance of codon mismatch is low under balanced conditions, accumulation of codon mismatch can lead to a high proportion of incorrect translation of long mRNAs (Fig 4a, 4b and 4c) and the chance can be increased under the Condition 2 (Fig 4b) and Condition 3 (Fig 4c). In Fig 4a, the fraction of incorrect peptide length does not change very much when there are more charged tRNA available, because under normal condition, the proportion of charged tRNAs can almost meet the demand of protein synthesis, and so is the balanced conditions of more charged tRNA available. So the fraction of mismatch on a codon almost remains unchanged (Fig 4d and 4f). But in Fig 4b, the fraction of incorrect peptide length increases fast with more unbalanced supplies. The reason is that when the number of Arginyl-tRNAs decreases or increases based on normal condition, the proportion of charged tRNAs cannot meet the demand of protein synthesis any longer. When the number of Arginyl-tRNAs decreases, the fraction of mismatch on Arg related codons will significantly increase, while the fraction of mismatch on other codons will decrease. When the number of Arginyl-tRNAs increases, the fraction of mismatch on Arg related codons will decrease, while the fraction of mismatch on other codons will increase (Fig 4e and 4g). Thus, study of codon mismatch effects is essential to build the model of the translation process.

### Mismatch is one reason of 5’-to-3’ ramp

**Fig 5:**
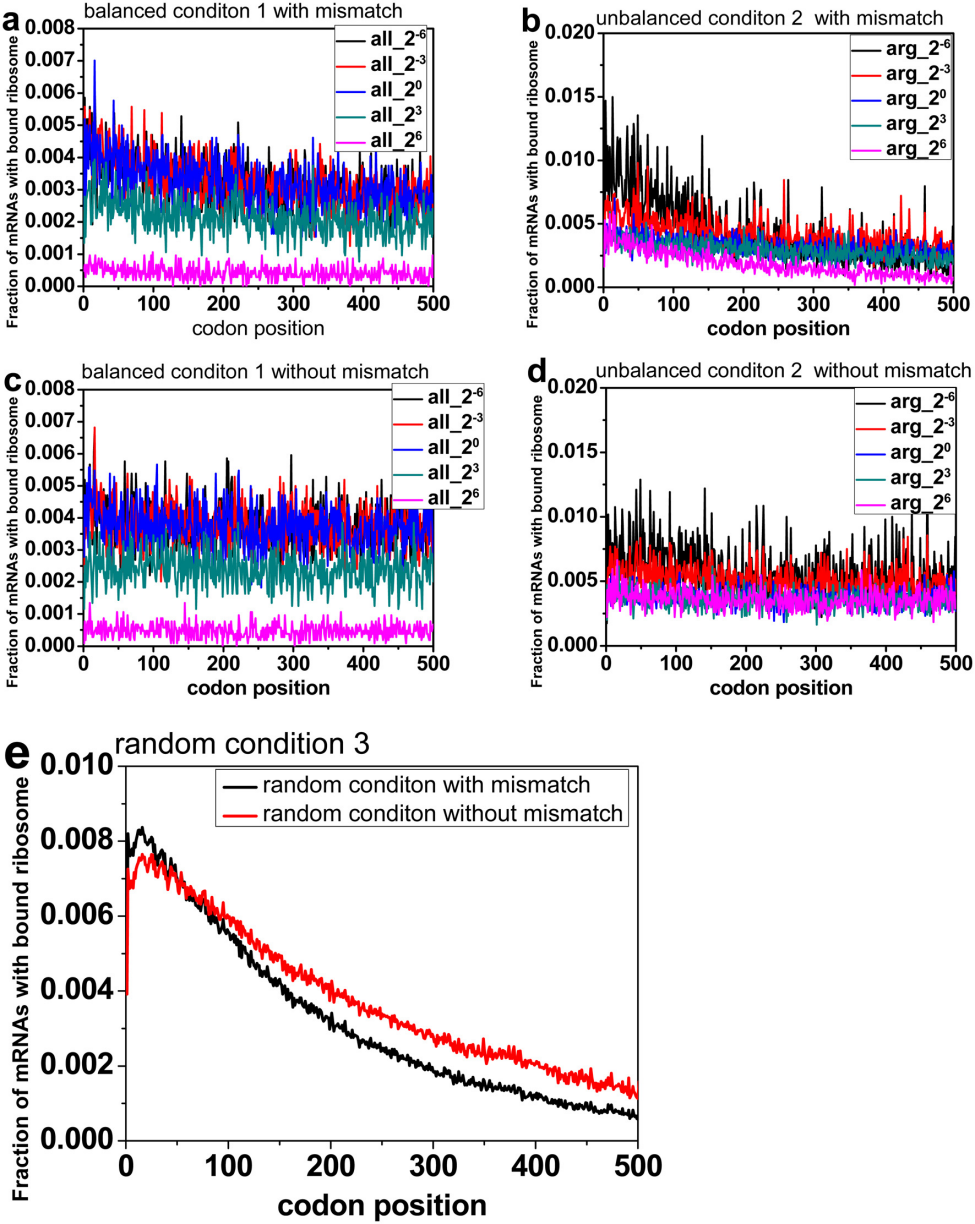
The effect of mismatch on 5’-to-3’ ramp. The vertical axis shows the fraction of mRNAs with bound ribosome. The horizontal axis shows the codon positions. Fraction of mRNAs with bound ribosome in each site was averaged over all the mRNAs that were larger than 500 codons. Fig 5a and 5b: simulation results with codon mismatch effects counted; Fig 5c and 5d: simulation results with no codon mismatch effects counted; Fig 5a and 5c: simulation results are under Condition 1; Fig 5b and 5d: simulation results are under Condition 2; Fig 5e: simulation results are under Condition 3 and the final fraction of mRNAs with bound ribosome of each site was averaged over the 200 simulations results.

Codon mismatch effects can be used to explain some well-known phenomenon in the translation process. In order to remove the effects of faster initiation of short mRNAs, 10410 mRNAs with codon sequence length larger than 500 codons were selected. To study the effect of mismatch on the 5’ ramp, the simulation with and without mismatch were run and the fraction of mRNAs with bound ribosome at each site was averaged over all the mRNAs. For each case (Fig 5a, 5b, 5c and 5d), a faster decline of fraction of mRNAs with bound ribosome is found through the elongation process when the mismatch events are considered (Fig 5b and 5d), which suggests that the mismatch premature events play an important role to form the 5’ ramp. The tests under Condition 3 with and without codon mismatch effects counted were also done (Fig 5e). There are 200 different random supply configurations and for each supply configuration, the simulation with and without codon mismatch effects counted were done. Finally the fraction of mRNAs with bound ribosome of each site was averaged over all the 200 simulations results. The result also shows that the curve declines faster when codon mismatch effects are counted.

Since the incorrect but complete translation would not affect the fraction of mRNAs with bound ribosome, it can be inferred that the fraction decrease along mRNA is, at least partly, caused by the premature termination mechanism. If a premature termination occurs, ribosome cannot translate the left codons, which will do contribution to a higher ribosome density in 5’ zones and lower ribosome density in 3’ zones.

In Fig 5, another observation is the lower fraction of mRNAs with bound ribosome when amino acids is more available, because when there are more charged aminoacyl tRNAs available, the ribosome will move faster from one codon to the next. But the initiation rate is the bottleneck for the whole translation process, which cannot be speeded as fast as the codon translation rate. So the increase of ribosome flux cannot be increased as fast as the increase of the charged aminoacyl tRNA,which will lead to a lower fraction of mRNAs with bound ribosome. Moreover, this effect is alleviated in Condition 2 under which only the number of Arginyl-tRNA fluctuates. When the number of Arginyl-tRNA increases, only the Arginyl-tRNA related codons will be affected significantly. That is to say only the ribosome at Arginyl-tRNA related codons site will translate faster than before, which cannot affect the fraction of mRNAs with bound ribosome very much. But when the Arginyl-tRNA drops in large number, all the codons will be affected because the ribosome on the Arginyl-tRNA related codons site moves slower, which will lead more stalled ribosomes, then the fraction of mRNAs with bound ribosome will increase significantly.

### Codon mismatch affects the rate of correct translation

**Fig 6:**
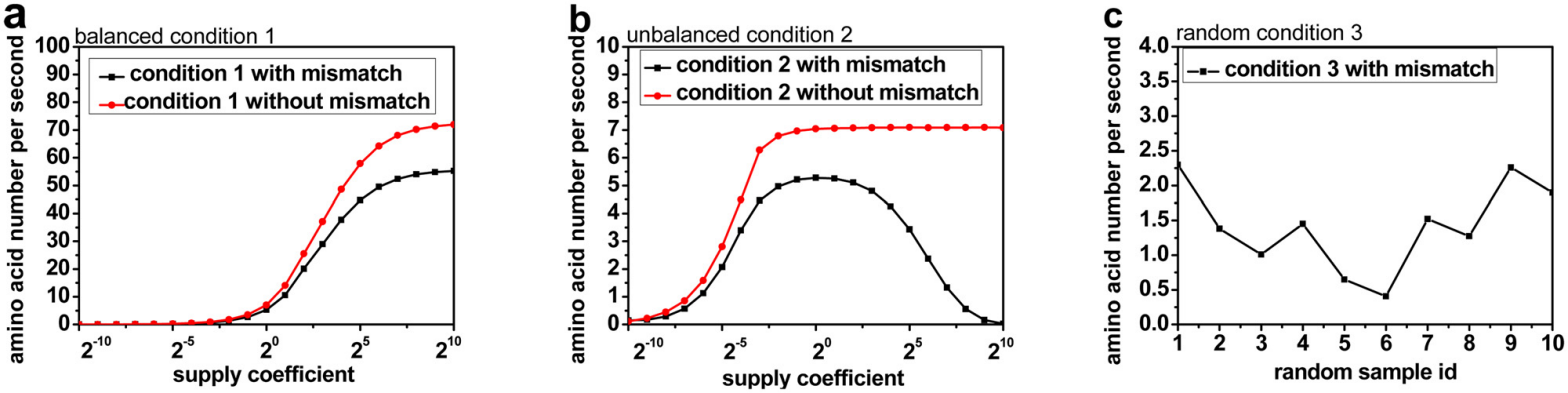
The effect of mismatch on the rate of correct translation. The vertical axis shows the average correct translation rate of one ribosome in a second. In Fig 6a and 6b, the horizontal axis shows the amino acids supply coefficient. The number of amino acid is equal to the normal condition number multiplied by the coefficient. So in Fig 6a, all amino acid numbers are multiplied by the same coefficient, and in Fig 6b, only the number of arg is multiplied by the coefficient, other amino acids keep unchanged. In Fig 6c, all the 10 simulations are under the Condition 3 that all amino acid supplies are randomly modified with the coefficient less than 1, and different amino acid has different coefficient.

Incorrect translation productions waste cell energies, decrease the rate of correct translation and even do harm to the living cell. The rate of the amino acid synthesis is about 5 aa/s under normal amino acid supply condition, which agrees with the results from empirical measurements[35,36]. In this work, the rates of correct translation under a variety of unbalanced amino acid supplies conditions were studied (Fig 6). It is interesting to see that the increase of Arg affects the translation rate in different manners. With a supply coefficient smaller than 2^0^, the translation rate increases with more Arg supplied due to the increase in reagent concentration. However, the trend is then reversed, which suggests that the degree of amino acid imbalance plays a more important role, causing a higher chance of codon mismatch and higher probability of incorrect translation. Thus, the codon mismatch is key to the determination of translation consequence.

## Discussion

All the above simulations are based on the assumption that amino acids are supplied continuously. But the actual environment is more complex than that. For example, there may be an extreme condition that the supply of some amino acids is ceased. As far as the model concerned, without the contribution of premature, there will be deadlock states on corresponding codon positions and the process of translation event will be paused without releasing the ribosome and the nascent peptide. From our results, it can be inferred that the premature termination can help avoid entering into the deadlock states. If cells release some incomplete nascent peptides through the premature mechanism and recover some scarce amino acids through the recycling mechanism[37–39], some of the stalled ribosomes caused by the deficient amino acids will continue to translate the left codons. Thus, although the codon mismatch does reduce the rate of valid work when amino acids supply is balanced, it can also be helpful when supply is unbalanced. Mismatch may be a way for living creatures to adapt to stressful environments. Due to the limitation of computational power and simulation algorithms, a number of simplifications were made in our model. The initialization, translation and termination should be each separated into several steps[40–42], which are not counted in this work. Additionally, in real biological environments the numbers of free tRNAs, ribosomes and mRNAs are changing continuously[25,43–45], while in our model these numbers are fixed. Some regulatory mechanisms[39,46,47] involved in the translation process are also ignored in the process of the simulation and other kinds of mismatch error that exist in the translation process[29,48] are also ignored. We mainly considered two supply conditions. One is balanced and the other is unbalanced. Considering the unbalanced supply of different amino acids have the similar effect, we just take the Arg as an example. In this model we have investigated the starvation condition that one amino acid is very deficient, but in fact, such a condition is terrible, almost no cell can keep live or cells have already adjusted the metabolic pathways to suit the terrible conditions. Based on the assumption that cells can survive and the amino acid metabolism pathway is not changed, we just only focus on the translation system behaviour and properties. It is more easier to get unusual behaviour under terrible conditions. Hopefully more parameters can be achieved in further experimental studies on various transition systems to improve this model.

## Supporting Information

S1 Appendix Supplement information of model. (DOC)
