# Supplementary material for: The Effect of Codon Mismatch on the Protein Translation System
Source: PLoS One. 2016 Feb 3;11(2):e0148302. doi: 10.1371/journal.pone.0148302 (PMC4739699; doi:10.1371/journal.pone.0148302)
Supplement: S1 Appendix — (DOC) [file pone.0148302.s001.doc]

# **Supplement information of model**

Dinglin Zhang^1¶^, Danfeng Chen^1¶^, Liaoran Cao^1^, Hong Cheng^2^, Guohui Li^1^,*

1.Pseudocode for AETASEP model

while current simulation time < total simulation time

{

Get initialization rate of each mRNA

Get elongation rate of each codon

Get the probability of each possible event based on its rate
Randomly select an event based on its probability
Update the state of the cell

if steps < Bsteps (basic steps for system equilibration)

Increase current simulation time and total simulation time by:$\Delta t=\frac{1}{\varepsilon^{t}+\rho^{t}}$

else

Increase current simulation time by: $\Delta t=\frac{1}{\varepsilon^{t}+\rho^{t}}$
Update the number of free ribosomes:$R^{f}$

}

2.Parameters

| $T_{real\_decay\_1/2}=432000s$ | Half life of ribosome (Nikolov EN 1983) |
| --- | --- |
| $t_{s}=0.2s$ | Peptide chain elongation step time (Karpinets TV, Greenwood DJ,2006) |
| $Bsteps=2\times10^{9}$ | basic steps for system equilibration(from the simulation) |
|  | mismatch probability between codon and anticodon(Zaher et al .2009) |
|  | Total abort probability after first mismatch for a ribosome in the left codon positions (Zaher et al .2009) |
| $s=7.78\times10^{\text{-4}}m$ | tRNA competition coefficient(Shah et al., 2013) |
| $R^{t}=200000$ | number of ribosomes (von der Haar, 2008; Warner, 1999) |
| $A^{t}=60000$ | number of mRNAs (Zenklusen et al., 2008) |
| $T^{t}=3300000$ | number of tRNAs (Waldron & Lacroute, 1975) |
| $T_{n}=41$ | number of types of tRNAs (Chan & Lowe, 2009) |
| $T_{j}^{t}=12000--190000$ | number of tRNAs of type $j$ (Chan & Lowe, 2009) |
| $A_{i}=1--1254$ | number of mRNA of type $i$ (Ingolia et al., 2009) |
| $p_{i}=3.5\times10^{-6}--0.115$ | gene-specific initialization probability (Shah et al., 2013) |
| $n=3795$ | number of genes (Ingolia et al., 2009) |
| $D_{r}=3.5\times10^{-13}m^{2}/s$ | Diffusion coefficient of ribosomes (Politz et al., 2013) |
| $D_{t}=3\times10^{-13}m^{2}/s$ | Diffusion coefficient of tRNAs (Werner, 2011) |
|  | size of ribosome footprint in codons (Ingolia et al., 2009) |
| $V=4.2\times10^{\text{-17}}m^{3}$ | volume of the cell (Jorgensen et al., 2002) |
|  | Characteristic time of ribosomes (Shah et al., 2013) |
|  | Characteristic time of tRNAs (Shah et al., 2013) |
|  | Discrete positions a ribosome can occupy in a yeast cell (Shah et al., 2013) |
|  | Discrete positions a tRNA can occupy in a yeast cell (Shah et al., 2013) |
| $\omega=0.64,0.61$ | Wobble parameter (Curran & Yarus, 1989; Lim & Curran, 2001) |

3.Mathematics analysis for codon mismatch effects

Because with the increase of the number of consecutive mistranslation, the probability will significantly reduce, so premature termination is assumed to occur immediately after the first mismatch with defined probability in this model.

The mismatch model can be described with the following formulas

　 (1)

　$\lambda=\ln(\frac{2}{t_{1/2}})$ (2)

$N_{t}$ is the number of the ribosome on the mRNA after time $t-t_{0}$, $N_{0}$is the number of the ribosome on the mRNA at $t_{0}$. $\lambda$ is a decay coefficient and $t_{1/2}$is the half-life of the aborted ribosome.

For one ribosome, the ribosome abort probability$p_{abort}$ after the one step time $t_{s}$ is :

(3)

$p_{mis\_abort}$is the total probability of premature on the left codons when mismatch occurs.

The probability for the ribosome to keep the binding state after one step time $t_{s}$ is:

(4)

The $t_{s}$ can be expressed as the ribosome elongation steps by approximation.

The ribosome can move about 2.8-10 codons in a second, so moving one code will take about 0.1-0.4 second. So $\lambda$ can be calculated based on different$p_{mismatch}$. Here we set $t_{s}=0.2s$, $p_{mis\_abort}=0.5$, then

$$\left\{\begin{matrix} 1:p_{mismatch}=0.01\to p_{abort}=0.005;p_{bind}=0.995;\lambda\approx0.025 \\ 2:p_{mismatch}=0.001\to p_{abort}=0.0005;p_{bind}=0.9995;\lambda\approx0.0025 \\ 3:p_{mismatch}=0.0001\to p_{abort}=0.00005;p_{bind}=0.99995;\lambda\approx0.00025 \\ 4:p_{mismatch}=0.00001\to p_{abort}=0.000005;p_{bind}=0.999995;\lambda\approx0.000025 \end{matrix}\right.$$

Scheme 1. After a period of simulation, the probability that the ribosome still binds on mRNA decreases due to codon mismatch. The black, red, blue and green lines represent the following *P* values respectively $p_{bind}=0.995$,$p_{bind}=0.9995$,$p_{bind}=0.99995$,$p_{bind}=0.999995$.

$$p_{bind}=0.999995$$

4.The effect of the ribosome real decay

The half-life of the ribosome is about 5 days[11].

$$T_{real\_decay\_1/2}=432000s$$

So $\lambda_{real\_decay\_1/2}=1.6\times10^{-6}$

The binding possibility after one step is

and the aborting possibility after one step is

We did not consider the decay of ribosome because the probability for decay is much smaller than the premature probability caused by codon mismatch.
